# The complete mitochondrial genome of *Tylototriton kweichowensis* and implications for *Tylototriton* taxonomy

**DOI:** 10.1080/23802359.2016.1180558

**Published:** 2016-09-04

**Authors:** Xiaonan Sun, Mei Ding, Ning Xiao, Kai Li, Tao Pan, Jiang Zhou, Baowei Zhang

**Affiliations:** a School of Life Sciences, Anhui University, Hefei, Anhui, China;; b Guiyang Nursing Vocational College, Guiyang, Guizhou, China;; c School of Life Sciences, Guizhou Normal University, Guiyang, Guizhou, China

**Keywords:** Mitochondrial genome, phylogenetic analysis, *Tylototriton kweichowensis*

## Abstract

In this paper, the complete 16,725 bp nucleotide sequence of the mitochondrial genome was determined for the *Tylototriton kweichowensis* (Caudata:Salamandridae). It contains 37 genes (13 protein-coding genes, 2 rRNA genes and 22 tRNA genes) and a non-coding region (D-loop). Overall base composition of the complete mitochondrial DNA is A (33.8%), G (14.4%), C (26.2%), and T (25.6%), so the percentage of A and T (59.4%) is higher than G and C (40.6%). All the genes in *T. kweichowensis* are distributed on the H-strand, except for the *ND6* subunit gene and eight tRNA genes which are encoded on the L-strand.

*Tylototriton kweichowensis* is a Class II State Major Protected Wildlife in China. It is found in western Guizhou and north-eastern Yunnan provinces. There is little information on the population of this species, but it is believed to be in decline. It inhabits low shrub and grass covered hills, however, breeding and larval development take place in pools and ponds (ICUN2016). The specimen was collected from Qianxi county of Guizhou Province (N27°00′, E106°02′) in August 201,5 which was provided by Jiang Zhou. Now the specimen was deposited in the laboratory of Evolution and Ecology, School of Life Sciences, Anhui University.

Total genomic DNA was extracted from muscle tissue using the standard phenol-chloroform protocol (Wang et al. 2010). The mitochondrial genome was amplified by polymerase chain reaction (PCR) using 15 pairs of primers. PCR products are sequenced directly in both directions. The mitochondrial genome was deposited in GeneBank after accurately annotated with the accession number KU320632. The complete mitochondrial genome sequence of *T. kweichowensis* is 16,725 bp in length and encodes 37 genes totally containing 13 PCGs and 22 tRNA genes, 2 rRNA genes and 1 control region. Among these, nine genes are encoded on the L strand, including *ND6* and eight tRNA genes; the remaining 28 genes are encoded on the H strand. The genes arrangement is similar to the complete mitochondrial genomes of other Salamandridae species (Zhang et al. 2008).

Phylogenetic relationships of 16 Tylototriton representative species are analyzed with Bayesian inference (BI) method using the MrBayes version 3.2 software (Ronquist et al. 2012) based on concatenated nucleotide sequences of the one protein-coding gene *ND2* of the mitochondrial genome, using *Echinotriton ander* as the out-group. In this process, the best-fitting nucleotide substitution model (GTR + I + G) was selected via MrModeltest version 2.1 (Nylander 2004); the Markov chain Monte Carlo (MCMC) was run with four chains for 1,000,000 generations until the average standard deviation of split frequencies reached a value less than 0.01, with Bayesian posterior probabilities calculated from the sample points after the MCMC algorithm had started to converge (Zhan & Fu 2011).

The phylogenetic tree is classified into Clade A, Clade B, Clade C and Clade D four major clades (Figure 1). The first lineage, Clade A, includes six species (*Tylototriton shanjing*, *T. verrucosus*, *T. uyenoi, T. yangi, T. kweichowensis* 1, *T. kweichowensis* 2).The second group, Clade B, includes *T. taliangensis*. The third lineage, Clade C, contains *T. liuyangensis*, *T. lizhenchangi*, *T. wenxianensis*, *T. dabienicus*, *T. vietnamensis*, *T. panha.* The last lineage, Clade D, is made up of *T. asperrimus*, *T. notialis* and *T. hainanensis*. Based on the results of our phylogenetic analyses, *T. kweichowensis* 1 and *T. kweichowensis* 2 are clustered into one clade which indicates that the species in our study is *T. kweichowensis*.

**Figure 1.**
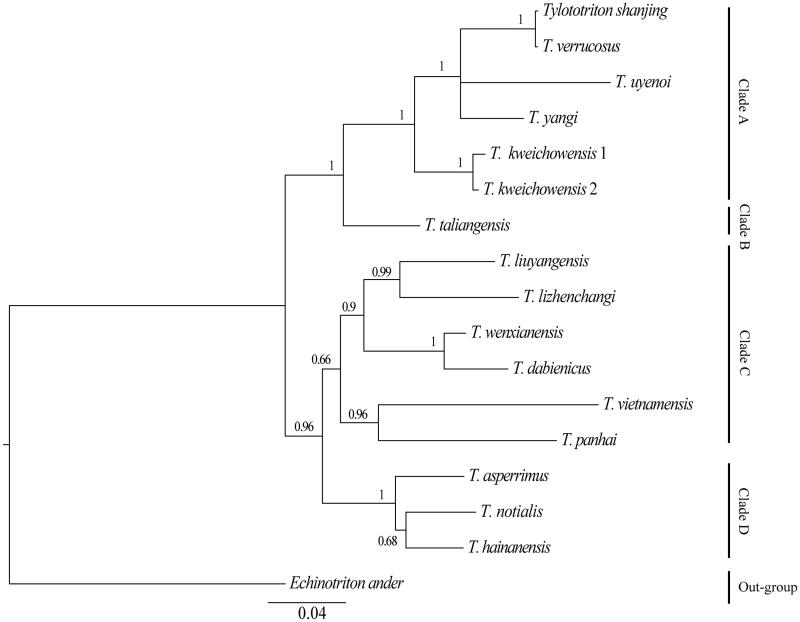
Inferred phylogenetic relationships among Tylototriton based on the nucleotide sequences of ND2 using Bayesian inference (BI). Numbers at each node indicate percentages of Bayesian posterior probabilities (BPPs). GenBank accession numbers for the published sequences are NC02750 (*Tylototriton wenxianensis*), JN934693 (*T. wenxianensis dabienicus*), KJ205598 (*T. liuyangensis*), AB769532 (*T. lizhenchangi*), AB769538 (*T. vietnamensis*), AB830737 (*T. panhai*), KC147816 (*T. asperrimus*), AB769536 (*T. notialis*), KC147817 (*T. hainanensis*), AB830727 (*T. shanjing*), AB830734 (*T. uyenoi*), AB830739 (*T. yangi*), NC02923 (*T. kweichowensis 1*), LC017835 (*T. verrucosus*), NC027421 (*T. taliangensis*), KU320632 (*T. kweichowensis* 2), NC017870 (*Echinotriton andersoni*).

Mitochondrial genome sequences have been proven to be useful for reconstructing phylogenetic relationship because of its small size, no intron, maternal inheritance, and contained much important phylogenetic information (Brown et al. 1979; Ballard & Whitlock 2004; Kumazawa & Endo 2004). We expect our result to provide a useful database for further studying the phylogenetic relationship of Salamandridae.
